# Clinical Outcomes and Urodynamic Effects of Tailored Transvaginal Mesh Surgery for Pelvic Organ Prolapse

**DOI:** 10.1155/2015/191258

**Published:** 2015-11-08

**Authors:** Ting-Chen Chang, Sheng-Mou Hsiao, Chi-Hau Chen, Wen-Yih Wu, Ho-Hsiung Lin

**Affiliations:** ^1^Department of Obstetrics and Gynecology, College of Medicine and National Taiwan University Hospital, National Taiwan University, 8 Chung-Shan South Road, Taipei 10041, Taiwan; ^2^Department of Obstetrics and Gynecology, Far Eastern Memorial Hospital, 21 Section 2, Nanya South Road, Banqiao District, New Taipei 20060, Taiwan

## Abstract

*Objective*. To evaluate the clinical outcomes and urodynamic effects of tailored anterior transvaginal mesh surgery (ATVM) and tailored posterior transvaginal mesh surgery (PTVM). *Methods*. We developed ATVM for the simultaneous correction of cystocele and stress urinary incontinence and PTVM for the simultaneous correction of enterocoele, uterine prolapse, vaginal stump prolapse, and rectocele. *Results*. A total of 104 women enrolled. The median postsurgical follow-up was 25.5 months. The anatomic cure rate was 98.1% (102/104). Fifty-eight patients underwent urodynamic studies before and after surgeries. The pad weight decreased from 29.3 ± 43.1 to 6.4 ± 20.9 g at 3 months. Among the 20 patients with ATVM, 13 patients had objective stress urinary incontinence (SUI) at baseline while 8 patients came to have no demonstrated SUI (NDSUI), and 2 improved after surgery. Among the 38 patients who underwent ATVM and PTVM, 24 had objective SUI at baseline while 18 came to have NDSUI, and 2 improved after surgery. Mesh extrusion (*n* = 4), vaginal hematoma (*n* = 3), and voiding difficulty (*n* = 2) were noted postoperatively. Quality of life was substantially improved. *Conclusions*. Our findings document the advantages of these two novel pelvic reconstructive surgeries for pelvic organ prolapse, which had a positive impact on quality of life. ATVM surgery additionally provided an anti-incontinence effect. This clinical trial is registered at ClinicalTrials.gov (NCT02178735).

## 1. Introduction

The estimated lifetime risk of surgery for either pelvic organ prolapse (POP) or stress urinary incontinence (SUI) in women is as high as 20% [1]. Among the different types of POP, anterior compartment prolapse is the most common and challenging to repair [2, 3]. Since the first publication of the vaginal repair of cystocele with a transobturator mesh in 2003 [4], various commercial kits have been developed. In particular, repair of cystocele with polypropylene mesh was found to result in less anatomical and symptomatic recurrent anterior prolapse than traditional colporrhaphy [5]. However, transobturator mesh systems have been reported to result in mesh extrusions and surgical reintervention at rates of 11.4% and 6.8%, respectively [5], as well as an increased incidence of de novo SUI [5]. Consequently, the use of mesh devices has come under increasing scrutiny by the US Food and Drug Administration because of concerns about complications [6].

Previously, our institute introduced a new method of cystocele repair using the purse-string technique reinforced with a custom-tailored, two-armed mesh [7]. Our transobturator mesh system uses a mesh with a smaller surface area than previous ones, resulting in fewer complications while maintaining low recurrence rates. Since 2011, we have modified our surgical procedures and developed two types of tailored transvaginal mesh (TVM) surgeries for the correction of POP: anterior TVM (ATVM) surgery for simultaneous correction of cystocele and SUI and posterior TVM (PTVM) surgery for simultaneous correction of enterocoele, uterine prolapse, vaginal stump prolapse, and rectocele. The purpose of this study was to evaluate the clinical outcomes and urodynamic effects of these two novel TVM surgeries.

## 2. Materials and Methods

A retrospective chart review of our hospital database was performed, including all patients treated for POP using TVM surgeries between November 2011 and November 2013. All procedures were performed by the same surgeons from the Department of Obstetrics and Gynecology at National Taiwan University Hospital. The study received institutional review board approval (protocol ID: 20140402RINC) and was registered at ClinicalTrials.gov (NCT02178735). The ethics committee waived the requirement for informed consent. The primary goal of this study was to evaluate anatomic success at 12 months after TVM surgery. Secondary outcomes included perioperative and long-term complication rates, as well as objective and subjective anti-incontinence success rates after TVM surgery.

The prolapse stage was defined according to the POP quantification (POP-Q) system [8]. Patients were evaluated during admission and reexamined at 6 weeks, 3 months, and 6 months and every year postoperatively. Each follow-up visit included a clinical examination and an interview concerning lower urinary tract symptoms and sexual functioning [8]. Any degree of descent ≥ stage 2 observed at a follow-up visit was considered a postoperative failure.

We have routinely performed preoperative urodynamic studies and pad testing in cases of cystocele to detect occult SUI [9, 10]. Patients were encouraged to undergo additional postoperative urodynamic studies and pad testing at the 3-month and 12-month follow-up visits to evaluate the effects of the TVM surgeries.

The Patient Perception of Bladder Condition Questionnaire, Overactive Bladder Symptom Score (OABSS) questionnaire [11], Urogenital Distress Inventory-6 (UDI-6) [12], Incontinence Impact Questionnaire-7 [12], King's Health Questionnaire [13], and POP/Urinary Incontinence Sexual Function Questionnaire (PISQ-12) [14] were translated into traditional Chinese and culturally adapted to Taiwanese society. A higher PISQ score represents a greater negative impact on sexual function.

### 2.1. Surgical Procedures

#### 2.1.1. ATVM

**Figure 1:**
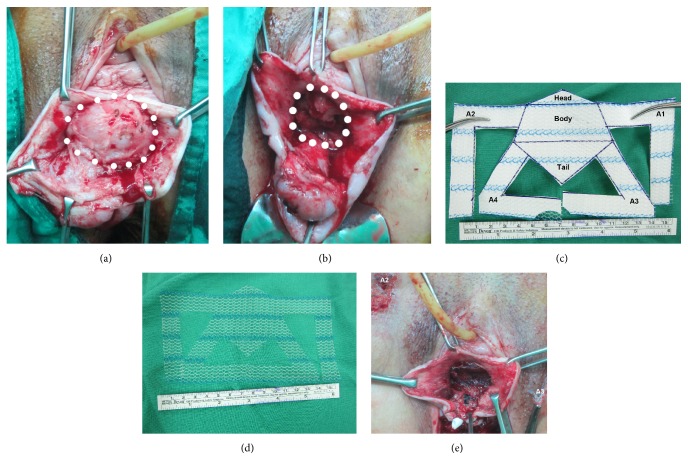
Components of the anterior transvaginal tailored mesh surgical procedure. (a) Separation of the bladder from the anterior vagina. White dotted circles indicate the area of the bladder base. (b) Use of the purse-string suture technique to reduce the cystocele size. The white dotted circles indicate the area of the reduced bladder base. (c) A drawing of the diamond body with its four arms and the polypropylene mesh (Gynemesh, 15 × 10 cm), which was trimmed according to the required shape. (d) Custom-tailored mesh with central diamond body and four arms. (e) Complete positioning of the body and arms of the mesh beneath the bladder, with the head of the diamond body underneath the bladder neck and proximal urethra without tension.

All surgical procedures were performed under intravenous general anesthesia (Figure 1). Every patient received a single dose of intravenous prophylactic antibiotic. The patients were placed in a dorsal lithotomy position, and the vaginal epithelium was injected with a vasoconstriction agent (20 units of Pitressin diluted in 80 mL of saline). The incision of the anterior vaginal wall was performed from the bladder base to the bladder neck and proximal urethra. After complete separation of the bladder from the vaginal wall, bilateral paravesical spaces were carefully opened (Figure 1(a)). Subsequently, a purse-string suture of the posterior bladder wall skipping the bladder neck was performed using Monocryl 2-0 (Figure 1(b)). A polypropylene mesh (Gynemesh, 15 × 10 cm) was then trimmed to a central diamond shape (7 × 6 cm) with two sets of paired arms (Figures 1(c) and 1(d)). In the next step of the procedure, two appropriate skin holes were created outside the left obturator foramen and then the right obturator foramen. A stainless steel tunneler was used to pull the A1 arm and then the A3 arm through to the left-side holes and subsequently the A2 and A4 arms through to the right-side holes using the outside-in method (Figure 1(d)). Specifically, a knot was tied at the tip of the stainless steel tunneler using a Vicryl 1-0 thread, and a needle was then used to suture one end of the thread to the end of a particular arm; the arm end and tunneler were then tied together, and we were able to pull each arm through the skin smoothly. The mesh was adjusted to the appropriate position under the bladder, and the tail of the diamond body was fixed by sutures (Surgilon 1-0) to the upper part of the anterior cervix. The cervical stump or vaginal cuff was pushed upward by adjusting the A3 and A4 arms on each side, and the head of the diamond body with arms A1 and A2 was adjusted to prevent excessive tension over the bladder neck. The right part of the mesh head was fixed to the right side of the periurethral tissue by sutures of Vicryl 2-0. The mesh was adjusted to prevent excessive tightness (Figure 1(e)), and the anterior vaginal wall was sutured in two layers without trimming the redundant portion using Vicryl 1-0.

#### 2.1.2. PTVM

**Figure 2:**
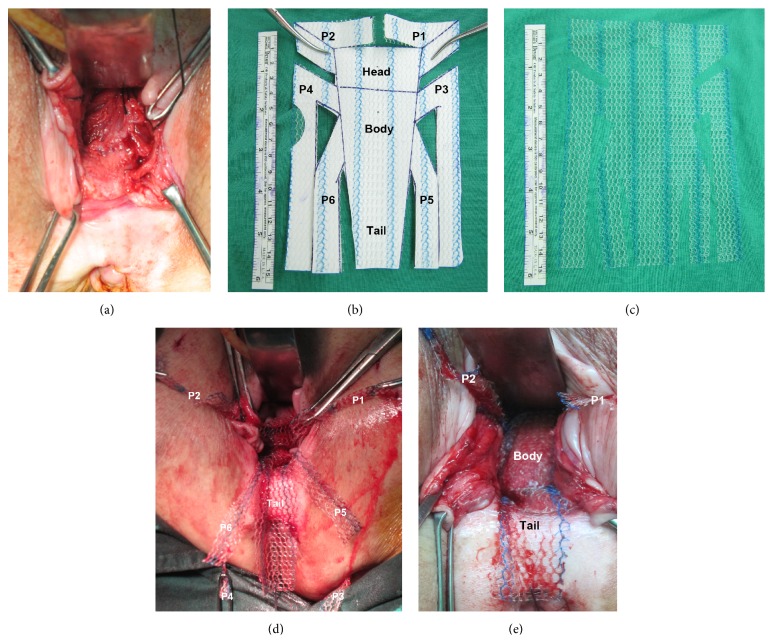
Components of the posterior transvaginal tailored mesh surgical procedure. (a) Separation of the rectum until the posterior fornix of the cervix is separated from the posterior vagina. (b) Drawing of the grasshopper body with its six arms and the polypropylene mesh (Gynemesh, 15 × 10 cm). (c) Custom-tailored mesh with the central grasshopper body and six arms. (d) Fixation of the head of the grasshopper-shaped portion to the posterior upper cervix and the insertion of the P1 and P2 arms into the bilateral uterosacral space without fixation. The P3 and P4 arms have already been inserted through the buttocks. (e) Complete positioning of the body and arms of the mesh covering the entire upper surface of the rectum without tension, with the exception of the P1 and P2 arms, which are to be inserted into the bilateral uterosacral spaces without fixation. The redundant tail part of the mesh will be trimmed to an appropriate length.

All surgical procedures were performed under endotracheal general anesthesia (Figure 2). An inverted T-shape incision of the posterior vaginal wall from the introitus to the posterior part of the cervix was performed after hydrodissection. Next, we separated the bilateral posterior vaginal wall from the rectum (Figure 2(a)) and carefully opened the bilateral pararectal spaces to identify the bilateral sacrospinous ligaments. One skin hole 3 cm lateral to and beneath the anal orifice was then created on the left buttock and again on the right buttock. Afterwards, the stainless tunneler, the tip of which was tied with several knots ~1 cm in length using Vicryl 1-0 thread, was used to penetrate the sacrospinous ligament, as guided by two fingers at each pararectal space, and then pulled through the P3 and then the P4 arms using the outside-in method (Figures 2(b), 2(c), and 2(d)). The forefinger and middle finger were then used to pull the two threads outside of the vagina. Using a needle to suture the end of each arm with one thread and tie them together, we were able to pull out each arm through the skin smoothly. With the same stainless steel tunneler, the P5 and P6 arms were sequentially pulled through the same skin holes on each side using the above-described procedure for ATVM. Next, the head of the mesh (half-folded) was fixed beneath the upper part of the cervix by sutures of Surgilon 1-0 (Figure 2(e)). The body of the mesh was then adjusted over the rectum by pushing the cervix upward with the fingers and simultaneously pulling arms P3–P6. Arms P1 and P2 were subsequently inserted into the bilateral uterosacral ligament spaces without fixation. The redundant portion of the mesh tail was trimmed while the lower portion of the mesh tail was fixed to the perirectal tissue using Vicryl 2-0 (Figure 2(e)). Finally, the posterior vaginal wall was sutured in two layers without trimming the redundant portion using Vicryl 1-0.

### 2.2. Urodynamic Assessment

A Life-Tech 6-channel urodynamic monitor with computer analysis software and Urovision Urolab Janus System V (Houston, TX, USA) were used for the urodynamic studies. A 20-minute pad test was also performed [10].

### 2.3. Statistical Analysis

STATA software (Version 11.0, Stata Corporation, College Station, TX, USA) was used for the statistical analysis. The Wilcoxon signed-rank test, Wilcoxon rank-sum test, and multivariate logistic regression analysis were used where appropriate.

Results are classified as either objective or subjective. Objectively, no demonstrated SUI (NDSUI) is defined as a finding of ≤1 g in the 20-minute pad test 3 or 12 months postoperatively [9, 10] while improvement is represented by a greater-than-50% decrease in pad weight compared to preoperative data 3 or 12 months postoperatively. Objective failure is indicated by a less-than-50% decrease in pad weight relative to preoperative data at the 3- or 12-month follow-up.

Subjective NDSUI is indicated by a score of zero on the third question of the UDI-6 questionnaire after surgery. Subjective SUI improvement is characterized by improvement on the third question of the UDI-6 questionnaire after surgery.

The diagnostic criteria for overactive bladder syndrome (OAB) included a score of 2 or higher for the third question of the OABSS questionnaire and a score of 3 or higher for the total score of the OABSS questionnaire [15]. The diagnostic criteria for urgency urinary incontinence included a score of 2 or higher for the fourth question of the OABSS questionnaire.

## 3. Results

A total of 104 consecutive patients were recruited between November 2011 and November 2013. Of these, 43 patients underwent ATVM alone while 8 had PTVM alone, and 53 underwent both ATVM and PTVM.  Table 1 contains the baseline characteristics of the sample. The median follow-up interval was 25.5 months (range: 15.5–40.2). The overall success rate for the sample was 98.1% (102/104).

The 96 patients who received ATVM had a cystocele stage ≥2. Although no postoperative failure was noted, four patients had mesh extrusion over the anterior vaginal compartment while two patients had voiding dysfunction, and two others had vaginal hematoma after ATVM surgery. All patients recovered well after the subsequent management.

All 61 patients who received PTVM had a rectocele, uterine prolapse, or vaginal stump prolapse stage ≥2. Two cases of postoperative failure occurred for the uterine prolapse surgery because of cervical elongation. In addition, one patient had vaginal hematoma but recovered well after subsequent management.

Urodynamic studies were performed for 58 patients who underwent ATVM, both before surgery and again 3 months postoperatively (Table 2). Among them, 31 patients (ATVM + PTVM, *n* = 22; ATVM, *n* = 9) received urodynamic studies 12 months postoperatively. The pad weight decreased significantly after surgery in both groups.

Among the above 20 patients with ATVM surgeries only, seven patients had NDSUI at baseline, and none developed de novo SUI after surgery. The other 13 patients had SUI at baseline while 8 patients came to have NDSUI, and 2 patients improved by 3 months after surgery (Table 2).

Among the above 38 patients with ATVM and PTVM surgeries, 14 patients had NDSUI at baseline, and two developed de novo SUI after surgery. The other 24 patients had SUI at baseline while 18 patients came to have NDSUI, and 2 patients improved by 3 months after surgery (Table 2).

Among the above 58 patients who underwent urodynamic studies, 55 patients had paired questionnaire data before and 3 months after surgery. Seventeen patients had subjective NDSUI at baseline, and 3 patients developed de novo SUI at 3 months after surgery. Among the 38 patients who had subjective SUI at baseline, 13 patients came to have NDSUI while 7 patients improved, 15 patients remained unchanged, and 3 became worse by 3 months after surgery.

Among the above 58 patients who underwent urodynamic studies, twenty-five patients had OAB at baseline, and 17 patients had OAB at 3 months after surgery (*P* = 0.09). Twenty-two patients had urgency urinary incontinence at baseline, and 12 patients had urgency urinary incontinence by 3 months after surgery (*P* = 0.01, Table 3).

Multivariate logistic backward stepwise regression analysis was performed using the preoperative pad weights and urodynamic parameters to predict postoperative objective and subjective NDSUI and improvement. Analyses yielded no significant predictors of either objective NDSUI or improvement. However, the pressure transmission ratio at maximum urethral pressure (odds ratio = 0.985, 95% CI = 0.972~0.999, and *P* = 0.034) predicted subjective NDSUI and improvement at 3 months after surgery, but not at 12 months. Meanwhile, the changes in pad weights were greater in our ATVM patients than the patients from our previous mesh procedure (mean: −22.8 versus 3.7 g, *P* = 0.005) (Table 4) [7]. Improvements were observed in the Patient Perception of Bladder Condition Questionnaire, OABSS questionnaire, UDI-6, Incontinence Impact Questionnaire-7, and most domains of King's Health Questionnaire at the 3-month and 12-month follow-up visits (Table 3).

## 4. Discussion

Using this innovative ATVM surgery for cystocele repair, we observed no cases of postoperative anatomic failure. This study confirms that the use of mesh at the time of anterior vaginal wall repair reduces the risk of recurrent anterior wall prolapse on examination [5]. Moreover, the changes in pad weight were significantly different between the present study and our previous research using the custom-tailored two-armed mesh [7], confirming the advantage of the anti-incontinence effect of this novel ATVM surgery.

To our knowledge, the anatomic cure rate of this novel ATVM surgery is superior to those of previous studies, which reported cure rates ranging from 75 to 100% [5, 7, 16, 17]. We consider this improvement to have resulted from the simultaneous correction of levels I, II, and III of the anterior vagina for cystocele [18].

Additionally, this procedure can simultaneously treat patients with SUI by using the head of the diamond body to cover the bladder neck and proximal urethra without tension. Moreover, the postoperative pad weight, maximum urethral pressure, and MUCP decreased simultaneously, indicating a different anti-incontinence mechanism of ATVM surgery, compared to the conventional midurethral sling procedure [19, 20].

Transobturator mesh systems are associated with an increased incidence of de novo SUI [5]. Because the obstructive effect of the prolapsed pelvic organs creates urethral kinking and increases urethral pressure, restoration of the anterior vaginal wall support may sacrifice urethral pressure dynamics, increasing the risk of postoperative SUI [21–23]. In our patients, the postoperative MUCPs were significantly lower compared to baseline, which may be due to the loss of preoperative urethral kinking after surgery. However, this adverse effect did not cause clinical problems in our patients because there was significant improvement in pad testing results. That is, our novel ATVM surgery provided an anti-incontinence effect, probably related to the novel design of the mesh head to support the bladder neck and proximal urethra, which is different from the anti-incontinence effect of the midurethral sling procedure. Our anti-incontinence result is also consistent with findings from two previous studies [24, 25]. However, Nauth and Fünfgeld placed their mesh at the midurethra level [24], and Sergent et al. anchored the horizontal arm of a T-shape mesh through the obturator space after an incision extending to 1 cm below the urethral meatus [25].

With respect to PTVM surgery, the two failure cases were due to cervical elongation rather than true descent of the uterine body and, therefore, may not constitute true cases of failure of this procedure. Both cases were treated with the Manchester procedure and had no long-term sequelae. Additionally, seven of 62 cases were treated for vaginal vault prolapse at a stage ≥2 and showed no recurrence. A possible reason for this very high success rate is that this procedure not only corrects levels I (upper part of the posterior cervix), II, and III [18] but also simultaneously fixes the sacrospinous ligament (with arms P3 and P4). To our knowledge, this is the first method of simultaneous correction of the defects of the posterior compartment of the vagina to achieve such highly successful outcomes [16, 26, 27]. However, it has been previously reported in the Cochrane reviews that the use of mesh for posterior vaginal wall prolapse should be avoided [5]; thus, a randomized controlled clinical trial is needed to confirm the advantage of our PTVM surgery.

Analyses of our quality of life data revealed high levels of satisfaction following both procedures, alone or in combination. Meanwhile, no significant decrease in PISQ-12 scores was observed. Mesh contraction is another disadvantage of transvaginal POP surgery with mesh, and mesh extrusion and mesh contraction can both lead to severe pelvic pain. Nevertheless, in our study no patient suffered from pelvic pain postoperatively.

Vaginal mesh extrusion is a common complication of transvaginal synthetic mesh placement for cystocele repair [21, 28]. We observed a relatively low rate (3.8%) of mesh extrusion with our tailored meshes compared to other published data (11~15.6%) [21, 28]. The low rate of mesh extrusion may be related to the significant experience of the surgeons in our sample, a decrease in the size of the bladder base using a purse-string suture, the tailored mesh used to provide the most appropriately sized central support beneath the bladder, a novel design of the mesh, and the usage of a double-layer suture of the vaginal mucosa and without trimming of any mucosa. However, some manufacturers have withdrawn their mesh kits following FDA warning, and some complex complications related to the use of mesh can be difficult to manage. Patient selection, a surgeon's skills, and proper training are all important when performing ATVM and PTVM surgeries. Larger studies are needed to confirm the outcomes of ATVM and PTVM surgeries noted here.

The study's retrospective design limits its generalizability. However, this study is also strengthened by the inclusion of sequentially pre- and postoperative urodynamic studies with pad testing in addition to questionnaires despite the presence of missing data.

## 5. Conclusions

This study presents our experiences with two novel TVM surgeries, each of which is feasible and utilizes safe procedures with good success rates. Overall, our findings reveal reasonable complication rates and better quality of life postoperatively. Additionally, the ATVM procedure also provided an anti-incontinence effect.

## Figures and Tables

**Table 1:** Baseline characteristics (*n* = 104).

Variables	ATVM (*n* = 43)	ATVM + PTVM (*n* = 53)	PTVM (*n* = 8)	*P* ^‡^
Age (years)	62.3 ± 10.5	64.2 ± 10.4	63.4 ± 12.3	0.59
Parity	3.1 ± 1.0	3.3 ± 1.4	2.6 ± 1.6	0.15
Menopause	36	46	8	0.68
Diabetes mellitus	5	14	2	0.15
Prior hysterectomy	11	7	2	0.22
Prior incontinence surgery	0	4	2	0.02
Prior prolapse surgery	2	6	0	0.43
Concomitant surgeries				
Midurethral sling procedure	0	0	6	<0.001
Miscellaneous	2	3	1	0.52
Operation time (minutes)	61.7 ± 21.8	118.5 ± 36.9	81.6 ± 16.9	<0.001
Blood loss (mL)	106.5 ± 129.4	133.2 ± 157.7	132.5 ± 86.3	0.23
Follow-up interval (months)	24.1 ± 7.4	21.0 ± 5.3	20.1 ± 5.8	0.06
Perioperative complications				
Vaginal hematoma	1	2	0	1.00
Voiding difficulty	2	0	0	0.32
Postoperative complications				
Mesh extrusion	1	3	0	0.73
Redundant anterior vaginal tissue	0	1	0	1.00
Postoperative failure				
Cystocele	0	0	0	—
Uterine prolapse ≥ stage 2	0	2	0	0.57
Rectocele	0	0	0	—

ATVM = tailored anterior transvaginal mesh surgery; PTVM = tailored posterior transvaginal mesh surgery. Values are expressed as the mean ± standard deviation or patient number.

^‡^By Kruskal-Wallis test, chi-square test, or Fisher's exact test.

**Table 2:** Changes in urodynamic variables and pad weights between baseline and after tailored anterior transvaginal mesh surgery (ATVM, *n* = 20) or combined ATVM and tailored posterior transvaginal mesh surgery (PTVM, *n* = 38).

Variables	ATVM					ATVM + PTVM				
	Baseline (a) *n* = 20	3 months (b) *n* = 20	12 months (c) *n* = 9	*P* ^‡^	*P* ^§^	Baseline (d) *n* = 38	3 months (e) *n* = 38	12 months (f) *n* = 22	*P* ^‡^	*P* ^§^
Pad weight (g)	23.7 ± 37.9	5.7 ± 14.4	10.9 ± 21.0	0.002	a versus b or c, *P* < 0.01	32.2 ± 45.9	6.7 ± 23.8	4.4 ± 10.5	<0.001	d versus e or f, *P* < 0.001
NDSUI	7	15	6	—	—	14	30	16	—	—
Improved	—	2	2	—	—	—	3	4	—	—
Failed	—	3	1	—	—	—	5	2	—	—
*Q*max (mL/s)	18.9 ± 11.3	19.9 ± 9.3	20.8 ± 8.6	0.83	—	21.4 ± 10.0	23.0 ± 9.6	22.0 ± 8.1	0.80	—
VV (mL)	287 ± 165	281 ± 116	267 ± 123	0.84	—	311 ± 136	306 ± 108	333 ± 151	0.56	—
PVR (mL)	42 ± 21	35 ± 14	32 ± 12	0.01	a versus b, *P* = 0.04	57 ± 32	35 ± 14	37 ± 22	<0.001	d versus e or f, *P* < 0.01
SD (mL)	245 ± 64	254 ± 47	262 ± 20	0.23	—	253 ± 55	266 ± 41	273 ± 36	0.08	—
Pdet*Q*max (cmH_2_O)	22.5 ± 15.9	23.8 ± 13.5	27.8 ± 21.0	0.69	—	22.0 ± 11.4	32.7 ± 35.5	28.5 ± 24.1	0.22	—
MCP (cmH_2_O)	98.2 ± 26.7	85.2 ± 23.3	89.1 ± 27.6	0.40	—	108.1 ± 30.9	85.8 ± 22.2	85.9 ± 27.7	<0.001	d versus e or f, *P* < 0.01
MUCP (cmH_2_O)	57.9 ± 24.9	41.5 ± 21.1	52.4 ± 25.4	0.007	a versus b, *P* = 0.002	64.8 ± 27.6	41.2 ± 20.6	45.8 ± 27.0	<0.001	d versus e or f, *P* < 0.01
FPL (cm)	2.7 ± 1.0	2.5 ± 0.7	2.4 ± 0.4	0.76	—	2.6 ± 0.6	2.4 ± 0.6	2.5 ± 0.7	0.15	—
PTR at MUP (%)	78 ± 29	95 ± 20	114 ± 40	0.002	a versus c, *P* = 0.008	123 ± 54	102 ± 39	110 ± 26	0.08	—
DO	5	2	0	—	—	2	0	0	—	—

The values are expressed as the mean ± standard deviation or patient number. DO = detrusor overactivity; FPL = functional profile length; MUCP = maximum urethral closure pressure; NDSUI = no demonstrated SUI; Pdet*Q*max = detrusor pressure at maximum flow rate; PTR at MUP = pressure transmission ratio at maximum urethral pressure; PVR = postvoid residual; *Q*max = maximum flow rate; SD = the volume at which a strong desire to void occurred; SUI = stress urinary incontinence; VV = voided volume.

^‡^Skillings-Mack test or McNemar's test.

^§^Post hoc test by Wilcoxon sign-rank test. Those without significant *P* values did not show here.

**Table 3:** The changes in the PPBC, OABSS, UDI-6, IIQ-7, King's Health Questionnaire, and PISQ-12 scores between baseline and after tailored anterior transvaginal mesh surgery (ATVM) or combined ATVM and tailored posterior transvaginal mesh surgery (PTVM) (*n* = 58).

Variables	Baseline (a) *n* = 58	3 months (b) *n* = 58	12 months (c) *n* = 19	*P* ^‡^	*P* ^§^
OAB	25	17	6	—	a versus b, *P* = 0.09
Urgency urinary incontinence	22	12	2	—	a versus b, *P* = 0.01; a versus c, *P* = 0.008
PPBC	3.5 ± 1.3	2.0 ± 1.1	2.1 ± 1.1	<0.001	a versus b, *P* < 0.001; a versus c, *P* = 0.006
OABSS	5.5 ± 3.8	3.9 ± 2.8	3.6 ± 2.7	0.004	a versus b, *P* < 0.004; a versus c, *P* = 0.009
UDI-6	6.2 ± 4.4	3.5 ± 3.6	3.1 ± 3.3	<0.001	a versus b, *P* < 0.001; a versus c, *P* = 0.002
IIQ-7	6.4 ± 5.8	2.23 ± 3.7	3.2 ± 5.0	<0.001	a versus b, *P* < 0.001; a versus c, *P* = 0.02

General health perception	50.4 ± 22.6	34.5 ± 21.2	36.8 ± 19.3	<0.001	a versus b, *P* < 0.001; a versus c, *P* = 0.003
Incontinence impact	38.7 ± 32.3	17.0 ± 24.7	22.8 ± 25.0	0.005	a versus b, *P* < 0.001; a versus c, *P* = 0.046
Role limitations	33.9 ± 31.6	14.2 ± 23.7	14.9 ± 20.7	<0.001	a versus b, *P* < 0.001; a versus c, *P* = 0.004
Physical limitations	39.3 ± 29.0	16.1 ± 22.9	18.4 ± 19.9	<0.001	a versus b, *P* < 0.001; a versus c, *P* = 0.003
Social limitations	22.9 ± 25.9	10.1 ± 18.6	10.2 ± 17.0	0.004	a versus b, *P* < 0.001; a versus c, *P* = 0.01
Personal relationships	18.8 ± 23.5	6.2 ± 11.5	8.3 ± 12.7	0.28	—
Emotions	33.3 ± 30.3	12.9 ± 20.3	16.4 ± 24.9	<0.001	a versus b, *P* < 0.001; a versus c, *P* = 0.047
Sleep/energy	34.2 ± 29.0	21.2 ± 22.6	20.2 ± 20.5	0.004	a versus b, *P* < 0.001; a versus c, *P* = 0.02
Severity measures	24.3 ± 27.7	13.9 ± 16.5	16.2 ± 25.7	0.07	—

PISQ-12 (*n* = 17)	15.6 ± 5.1	14.1 ± 4.1	—	0.19	—

The values are expressed as the mean ± the standard deviation or patient number.

^‡^Skillings-Mack test or Wilcoxon signed-rank test (PISQ-12).

^§^Post hoc test by Wilcoxon sign-rank test. Those without significant *P* values did not show here.

IIQ-7 = Incontinence Impact Questionnaire-7; OAB = overactive bladder syndrome; OABSS = Overactive Bladder Symptom Score; PISQ-12 = Pelvic Organ Prolapse/Urinary Incontinence Sexual Function Questionnaire; PPBC = Patient Perception of Bladder Condition Questionnaire; UDI-6 = Urinary Distress Inventory-6 Questionnaire.

**Table 4:** Linear regression analysis of the change from baseline with this novel surgery compared to our prior study [7].

Variables	Baseline		Change from baseline at 3 months		
	This study (*n* = 58)	Prior study [7] (*n* = 21)	This study (*n* = 58)	Prior study [7] (*n* = 21)	*P* ^‡^
Pad weight (g)	29.3 ± 43.1	5.9 ± 14.6	−22.9 ± 38.5	3.7 ± 16.3	0.005
*Q*max (mL/s)	20.5 ± 10.4	22.3 ± 12.0	3.9 ± 22.2	−0.6 ± 11.0	0.38
VV (mL)	302 ± 146	350 ± 129	−5 ± 164	−19 ± 123	0.73
PVR (mL)	51.4 ± 29.4	100.7 ± 59.9	−16.3 ± 29.4	−13.4 ± 80.9	0.82
SD (mL)	250 ± 58.0	307 ± 78	12 ± 51	−1.8 ± 90.4	0.39
Pdet*Q*max (cmH_2_O)	22.2 ± 12.9	27.6 ± 17.6	8.4 ± 30.2	−1.7 ± 16.9	0.17
MUP (cmH_2_O)	104.7 ± 29.7	103.0 ± 27.3	−19.1 ± 27.1	−20.7 ± 28.7	0.63
MUCP (cmH_2_O)	62.4 ± 26.6	70.4 ± 24.7	−21.1 ± 27.3	−21.3 ± 24.5	0.97
FPL (cm)	2.6 ± 0.7	3.4 ± 0.8	−0.2 ± 0.9	0.2 ± 1.3	0.12
PTR at MUP (%)	108 ± 51	101 ± 48	−9 ± 54	−14 ± 48	0.68
DO^§^	7	6	2	8	0.58

The values are expressed as the mean ± standard deviation or patient number.

^‡^Wilcoxon rank-sum test.

^§^McNemar's test.

Abbreviations are the same as in Table 2.
